# HbA1c as screening for gestational diabetes mellitus in women with polycystic ovary syndrome

**DOI:** 10.1186/s12902-015-0039-9

**Published:** 2015-08-06

**Authors:** Ingrid Hov Odsæter, Arne Åsberg, Eszter Vanky, Sven Magnus Carlsen

**Affiliations:** Department of Clinical Chemistry, St. Olavs Hospital, Trondheim University Hospital, Trondheim, Norway; Department of Obstetrics and Gynecology, St. Olavs Hospital, Trondheim University Hospital, Trondheim, Norway; Department of Endocrinology, St. Olavs Hospital, Trondheim University Hospital, Trondheim, Norway; Department of Cancer Research and Molecular Medicine, Faculty of Medicine, Norwegian University of Science and Technology, Trondheim, Norway; Department of Laboratory Medicine, Children’s and Women’s Health, Trondheim, Norway; Unit for Applied Clinical Research, Faculty of Medicine, Norwegian University of Science and Technology, Trondheim, Norway

**Keywords:** Gestational diabetes mellitus, Polycystic ovary syndrome, HbA1c, Preeclampsia, Birth weight

## Abstract

**Background:**

Gestational diabetes mellitus (GDM) is associated with adverse pregnancy outcomes such as preeclampsia and macrosomia. Women with polycystic ovary syndrome (PCOS) are at increased risk of developing GDM. Today, GDM is diagnosed by oral glucose tolerance test (OGTT), a rather cumbersome test for the women and health care system. The objectives of this study were to investigate whether HbA1c in first trimester of pregnancy could be used as a screening test for GDM in first trimester and throughout pregnancy in order to reduce the number of OGTTs, and whether it could predict preeclampsia and macrosomia in women with PCOS.

**Methods:**

Post hoc analyses of data from 228 women from a prospective, randomised, multicenter study comparing metformin to placebo from first trimester to delivery. Fasting and 2-h plasma glucose were measured during a 75 g OGTT in first trimester, gestational week 19 and 32 as well as fasting plasma glucose in gestational week 36. GDM was diagnosed by WHO criteria from 1999 in first trimester and throughout pregnancy and by *modified* IADPSG criteria (i.e. lacking the 1-h plasma glucose value) in first trimester. The diagnostic accuracy was assessed by logistic regression and ROC curve analysis.

**Results:**

The area under the ROC curve for first trimester HbA1c for screening of GDM diagnosed by WHO criteria in first trimester was 0.60 (95 % CI 0.44-0.75) and 0.56 (95 % CI 0.47-0.65) for GDM diagnosed throughout pregnancy. Only 2.2 % (95 % CI 0.7-5.1 %) of the participants could have avoided OGTT. HbA1c was not statistically significantly associated with GDM diagnosed by *modified* IADPSG criteria in first trimester. However, first trimester HbA1c was statistically significantly associated with preeclampsia. Both HbA1c and GDM by WHO criteria in first trimester, but not by IADPSG, were negatively associated with birth weight.

**Conclusion:**

First trimester HbA1c can not be used to exclude or predict GDM in women with PCOS, but it might be better to predict preeclampsia than the GDM diagnosis.

**Electronic supplementary material:**

The online version of this article (doi:10.1186/s12902-015-0039-9) contains supplementary material, which is available to authorized users.

## Background

Gestational diabetes mellitus (GDM) is defined as hyperglycemia first detected in pregnancy that is less severe than diabetes mellitus in non-pregnant adults [1]. Women with GDM are at increased risk of adverse pregnancy outcomes, especially preeclampsia and macrosomia of the newborn [1]. Today, several sets of diagnostic criteria exist for GDM and a common feature is that they are based on an oral glucose tolerance test (OGTT) [2]. These tests are time-consuming, require overnight fasting and some women become nauseous after drinking the glucose solution. Glycated hemoglobin A1c in blood (HbA1c), a marker of chronic glycaemia, has been approved as a diagnostic test for diabetes mellitus in non-pregnant women [3]. It has several advantages compared to OGTT, as fasting, glucose ingestion and timed samples are not required, and the preanalytical instability is less [3]. In addition, the analysis is now well standardised [3].

Several studies on the possible role of HbA1c in screening for GDM have been published [4–10], where HbA1c typically has been measured in late second or third trimester. The results from most of the studies are consistent with HbA1c not being a suitable screening test for GDM. Recently, Fong et al. found that women with HbA1c of 5.7-6.4 % at first prenatal visit (up to 20 weeks of gestation) had a 3-fold higher risk of developing GDM compared to those with HbA1c < 5.7 % [11]. The result remained statistically significant when the analysis was restricted to those with HbA1c measured in first trimester.

Polycystic ovary syndrome (PCOS) is characterised by oligo-amenorrhea, hyperandrogenism and polycystic ovaries. According to the Rotterdam criteria, at least two of the three characteristics must be present to diagnose the syndrome [12]. PCOS is considered to be the most common endocrine disorder among women of fertile age. In a Norwegian study, the prevalence of PCOS according to the Rotterdam criteria was 14.2 % in women with previous term deliveries [13]. In a cross-sectional study of 392 adult Turkish women the prevalence was 19.9 % [14], and 17.8 % in a birth cohort-based study of Australian women [15]. Women with PCOS have a higher prevalence of impaired glucose tolerance and type 2 diabetes mellitus when compared to BMI-matched women not having PCOS [16]. They also have an increased risk of developing GDM [17–20]. A prevalence of GDM of 25.6 % and 24.2 % by the 1999 World Health Organization (WHO) criteria and *modified* (i.e. lacking the 1-h plasma glucose value) International Association of the Diabetes and Pregnancy Study Groups (IADPSG) criteria, respectively, has been reported in a Norwegian PCOS population [21]. It is widely held that in many women GDM is an early manifestation of type 2 diabetes mellitus, and it is suggested that women at increased risk of diabetes should be tested for hyperglycemia early in pregnancy [22]. As far as we know, no study on HbA1c in pregnant women with PCOS has been published. The primary aim of this study was to investigate whether first trimester HbA1c in women with PCOS could be used to screen for GDM in first trimester and GDM diagnosed throughout pregnancy, in order to reduce the number of OGTTs. A secondary aim was to study whether first trimester HbA1c could predict preeclampsia and macrosomia.

## Methods

### Study design

The current study used data from the previously reported PregMet (Metformin treatment in pregnant women with PCOS) study which was a prospective, randomised, double-blind, multicentre trial in women with PCOS comparing 2000 mg metformin daily against placebo from first trimester to delivery [23]. Pregnant women with PCOS aged 18-45 years with a singleton viable fetus between gestational week 5 and 12 were included. PCOS was diagnosed according to the Rotterdam criteria before the actual pregnancy [12]. Exclusion criteria were alanine aminotransferase > 90 IU/L, creatinine > 130 μmol/L, known alcohol abuse, previously diagnosed diabetes mellitus or fasting plasma or serum glucose > 7.0 mmol/L at inclusion, treatment with oral glucocorticoids or use of drugs known to interfere with metformin.

In the PregMet study, pregnant women were recruited from gynaecological outpatient clinics, fertility clinics, private practice and because of referral to an ultrasound early in pregnancy. The participants were enrolled from February 2005 until January 2009 at 11 study centres (three university hospitals, seven local hospitals and one gynaecological specialist practice) in Norway.

At inclusion, gestational week 19 and 32 a 75 g 2-h OGTT was performed according to the recommendations of the WHO [24]. At gestational week 36, fasting plasma or serum glucose was measured. Those already diagnosed with GDM did not perform a new OGTT, only fasting plasma or serum glucose was measured at subsequent time points. All participants received written and verbal diet recommendations according to the general guidelines for all pregnant women in Norway. Those diagnosed with GDM received more thorough diet and lifestyle advice. Insulin treatment was considered if plasma glucose levels 1–1.5 h after meal were > 8 mmol/L. Only two of the study participants required insulin treatment.

All study participants gave a written informed consent before inclusion. The Committee for Medical Research Ethics of Health Region IV, Norway, and The Norwegian Medicines Agency approved the study. The Declaration of Helsinki was followed throughout the study and the study was conducted according to principles of Good Clinical Practice. The study is registered at www.clinicaltrials.gov as NCT00159536.

### Diagnostic criteria

GDM was diagnosed according to the WHO criteria from 1999 as fasting plasma or serum glucose ≥ 7.0 mmol/L or plasma or serum glucose ≥ 7.8 mmol/L two hours after ingesting 75 g glucose orally (OGTT) [24]. Due to the exclusion of women with fasting plasma or serum glucose > 7 mmol/L, only participants with a 2-h OGTT result above the diagnostic cut-off value at inclusion were diagnosed as having GDM *in first trimester*. In addition, participants with at least one result above a diagnostic cut-off value at inclusion, gestational week 19, 32 or 36 were diagnosed as having GDM *throughout pregnancy*. We also diagnosed GDM *in first trimester* according to *modified* IADPSG criteria as fasting plasma or serum glucose ≥ 5.1 mmol/L or plasma or serum glucose ≥ 8.5 mmol/L two hours after the glucose load [22].

GDM diagnosed by the WHO criteria will be named GDM-WHO, and participants not diagnosed with GDM-WHO will be described as normal glucose tolerant (NGT-WHO). GDM diagnosed by *modified* IADPSG criteria will be named GDM-IADPSG, and those not diagnosed with GDM-IADPSG will be called NGT-IADPSG.

Preeclampsia was diagnosed as blood pressure of 140/90 mm Hg or higher measured on two occasions after gestational week 20 and albuminuria of at least + 2 dipstick on one occasion or + 1 dipstick on two occasions.

### Laboratory analyses

Plasma or serum glucose was analysed in venous blood samples drawn from an antecubital vein between 08 and 11 am after an overnight fast. Thereafter a 75 g OGTT was performed and two hours later a blood sample was drawn. The samples were collected and processed in accordance with local standardised procedures at the participating study centres.

HbA1c was not analysed during the study period. Venous blood samples collected in EDTA tubes were stored and available from 228 of the participants from inclusion. These samples had been stored at -80 °C for four to eight years before analysis. HbA1c is stable at these storage conditions [25, 26]. The samples were analysed in four runs at our hospital laboratory (St. Olavs Hospital, Trondheim University Hospital) by an immunoturbidimetric assay on a Roche Cobas Integra 400 + instrument (Roche Diagnostics, Mannheim, Germany) [27]. The results are traceable to the reference method of the International Federation of Clinical Chemistry and Laboratory Medicine [28]. The analyses were performed during a four-week period. The analytical coefficient of variation from day-to-day in this period was 0.8 % at HbA1c level 5.6 % (38 mmol/mol) and 1.0 % at HbA1c level 9.1 % (76 mmol/mol).

### Statistical analyses

We used logistic regression with backwards elimination to find the best combination of variables in predicting GDM-WHO in first trimester, GDM-IADPSG in first trimester, GDM-WHO throughout pregnancy and preeclampsia. The diagnostic accuracy of the various test combinations were assessed by receiver operating characteristic (ROC) curve analysis [29]. To find the best combination of variables predicting birth weight, we used multiple linear regression. For all the regression analyses we used the Royston and Altman algorithm to find the simplest (if any) non-linear transformation of the continuous variables [30]. The selection of variables considered for the models was based on variables that should be more easily available to the clinician in the first trimester than HbA1c, and that are known to be associated with or suspected to influence on the dependent variable. The significance level for keeping variables in the model was set at 0.10.

Predictor variables considered for the models for GDM in first trimester were HbA1c, age and body mass index (BMI) at inclusion, GDM in previous pregnancy and using metformin at conception (i.e. using metformin at conception and in early pregnancy followed by a washout period of at least 7 days before inclusion in the study). The same variables were considered for the model predicting GDM-WHO throughout pregnancy in addition to using metformin during pregnancy.

Predictor variables considered for the preeclampsia model were HbA1c, age and BMI at inclusion, using metformin at conception, using metformin during pregnancy, GDM-WHO in first trimester, nulliparity, smoking in first trimester (as a dichotomous variable), preeclampsia in previous pregnancy and pre-gestational hypertension. In addition, we performed the analyses with GDM-IADPSG in first trimester as an independent variable instead of GDM-WHO in first trimester.

The following predictor variables were considered for the model predicting birth weight; HbA1c, age and BMI at inclusion, using metformin at conception, using metformin during pregnancy, GDM-WHO in first trimester, nulliparity and smoking in first trimester (as a dichotomous variable). In addition, we performed the analyses with GDM-IADPSG in first trimester as an independent variable instead of GDM-WHO in first trimester.

The level of statistical significance was set at p-value 0.05. MedCalc version 12.0 for Windows (MedCalc Software, Ostend, Belgium) was used to calculate confidence interval for a rate. The other statistical analyses were performed using Stata version 13.1 for Windows (Stata Corp., Texas, USA).

## Results

Three hundred forty-eight women with a total of 364 pregnancies were considered eligible, whereof 90 were excluded: 58 declined to participate and 32 did not meet inclusion criteria. Two hundred seventy-four pregnancies (in 258 women) were included. Eighteen women were included later than intended, i.e. between gestational week 13 and 15. One woman was later excluded due to a partial 21-hydroxylase deficiency that was initially missed. Sixteen women participated twice. In the present post hoc analyses, we excluded the data from the second participation from these women, to avoid dependencies in the data that may bias the results. Women with no HbA1c result at inclusion (N = 29) were also excluded. In total, 228 women were included in these post hoc analyses, i.e. 66 % of those considered eligible. Twelve women dropped out, eight immediately after inclusion and four after gestational week 24. Data on pregnancy outcomes were available for these women and they were included in the analyses.

### Population characteristics and incidence of GDM

**Fig. 1:**
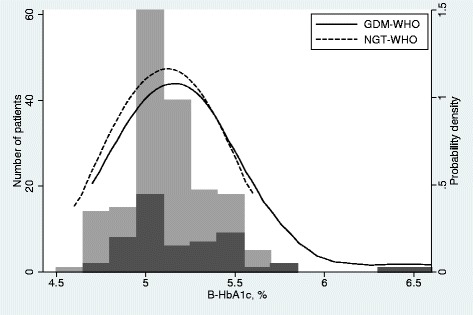
The distribution of HbA1c in those diagnosed with gestational diabetes mellitus and not (throughout pregnancy) by the 1999 WHO criteria. In addition to the histograms, the figure shows a kernel density plot of HbA1c in each group, where the distributions are smoothed and scaled to the same level of probability density

Of the 228 included women, 55 (24.1 %) developed GDM-WHO. Twenty women (8.8 %) had GDM-WHO at inclusion, another 21 (9.2 %) were diagnosed in gestational week 19 and 13 (5.7 %) were diagnosed in gestational week 32. In addition, one (0.4 %) was diagnosed in gestational week 36 because of an elevated fasting plasma glucose level. Thirty-five women were diagnosed with GDM-IADPSG at inclusion (15.4 %). Characteristics of the study population are given in Table 1, and the distribution of HbA1c in those with GDM-WHO and NGT-WHO throughout pregnancy is shown in Fig. 1.

**Table 1:** Characteristics of the study population

Characteristic	N	Median (min-max) or n (%)
Age (years)	228	30 (19-44)
BMI (kg/m^2^)	228	27.3 (18.8-50.2)
Caucasian (no.)	228	221 (96.9 %)
GDM in previous pregnancy (no.)	228	10 (4.4 %)
Preeclampsia in previous pregnancy (no.)	228	8 (3.5 %)
Pregestational hypertension (no.)	227	11 (4.9 %)
Smoking in first trimester (no.)	227	16 (7.1 %)
Nulliparity (no.)	228	132 (58 %)
Metformin use at conception (no.)	228	73 (32 %)
Randomised to metformin (no.)	228	114 (50 %)
Preeclampsia (no.)	228	14 (6.1 %)
Birth weight (g)	227	3550 (165-4840)
HbA1c in first trimester (%, (mmol/mol))	228	5.1 (4.6-6.6) (32 (27-49))
Fasting glucose in first trimester (mmol/L)	228	4.6 (3.4-6.6)
2-h glucose in first trimester (mmol/L)	226	5.4 (2.5-10.4)
Fasting glucose in gestational week 19 (mmol/L)	213	4.3 (3.4-6.6)
2-h glucose in gestational week 19 (mmol/L)	198	5.7 (2.4-10.7)
Fasting glucose in gestational week 32 (mmol/L)	202	4.4 (3.1-9.4)
2-h glucose in gestational week 32 (mmol/L)	177	6.2 (2.5-9.8)
Fasting glucose in gestational week 36 (mmol/L)	194	4.3 (3.4-9.2)

### First trimester HbA1c as a screening test for GDM

HbA1c (OR 7.6 per % increase in HbA1c, 95 % CI 1.37-42, p = 0.02) and age (OR 1.20 per year increase in age, 95 % CI 1.07-1.35, p = 0.002) were the only significant predictor variables in the model for GDM-WHO in first trimester, see Table 2. The area under the ROC curve for this model was 0.75 (95 % CI 0.63-0.88), and 0.60 (95 % CI 0.44-0.75) for HbA1c alone. Only one woman had an HbA1c below the cut-point that would give 100 % sensitivity (she had an HbA1c of 4.6 % (27 mmol/mol)), and only four women had an HbA1c above the cut-off level giving 100 % specificity (i.e. HbA1c > 5.6 % (38 mmol/mol)), see Table 3. Therefore, only five of the 228 patients (2.2 %, 95 % CI 0.7-5.1 %) could have avoided an OGTT if we had used these cut-off levels.

**Table 2:** Odds ratio, 95 % confidence interval and p-value for predictors for GDM-WHO in first trimester, GDM-IADPSG in first trimester, GDM-WHO throughout pregnancy and preeclampsia

	GDM-WHO in first trimester			GDM-IADPSG in first trimester^a^			GDM-WHO throughout pregnancy			Preeclampsia		
Predictor	OR	95 % CI	*p*-value	OR	95 % CI	*p*-value	OR	95 % CI	*p*-value	OR	95 % CI	*p*-value
HbA1c	7.6	1.37-42	0.02				3.1	0.87-11	0.08	17	2.4-115	0.004
Age	1.20	1.07-1.35	0.002	1.10	1.01-1.19	0.03	1.10	1.02-1.18	0.01			
GDM in previous pregnancy							3.2	0.85-12	0.08			
Preeclampsia in previous pregnancy										27	2.0-353	0.01
Nulliparity										7.7	0.97-62	0.054

^a^Modified IADPSG criteria were used, i.e. the 1-h plasma glucose value was missing

**Table 3:** Performance parameters for first trimester HbA1c in screening for gestational diabetes mellitus diagnosed by 1999 WHO criteria in first trimester and throughout pregnancy

Diagnosis	Cut-off level for HbA1c for dropping OGTT, % (mmol/mol)	Sensitivity, %	Specificity, %	Number of women avoiding OGTT	Number of women avoiding OGTT incorrectly classified
GDM-WHO in first trimester	<4.7 (28)	100	0.5	1	0
	<4.8 (29)	95.0	2.4	6	1
	<4.9 (30)	95.0	7.8	17	1
	>5.4 (36)	30.0	93.7	19	13
	>5.5 (37)	25.0	97.6	10	5
	>5.6 (38)	20.0	100	4	0
GDM-WHO throughout pregnancy	<4.7 (28)	100	0.6	1	0
	<4.8 (29)	98.2	2.9	6	1
	<4.9 (30)	96.4	8.7	17	2
	>5.4 (36)	14.6	93.6	19	11
	>5.5 (37)	9.1	97.1	10	5
	>5.6 (38)	7.3	100	4	0

Only higher age was associated with GDM-IADPSG in first trimester (OR 1.10 per year increase in age, 95 % CI 1.01-1.19, p = 0.03), see Table 2.

Predictor variables included in the model for GDM-WHO throughout pregnancy were HbA1c at inclusion (OR 3.1 per % increase in HbA1c, 95 % CI 0.87-11, p = 0.08), GDM in previous pregnancy (OR 3.2, 95 % CI 0.85-12, p = 0.08) and age (OR 1.10 per year increase in age, 95 % CI 1.02-1.18, p = 0.01), see Table 2. The area under the ROC curve of this combination of variables in diagnosing GDM-WHO throughout pregnancy was 0.65 (95 % CI 0.56-0.73), while with HbA1c as the only discriminator the area under the ROC curve was 0.56 (95 % CI 0.47-0.65). The same five participants as mentioned in the previous section on GDM diagnosed in first trimester only, could have avoided an OGTT by screening with HbA1c in first trimester for GDM-WHO throughout pregnancy if we had required 100 % sensitivity and 100 % specificity to exclude and to predict GDM, respectively, see Table 3.

We did not find any non-linear association between the possible continuous predictor variables and the outcome variables.

### First trimester HbA1c as a predictor for preeclampsia and birth weight

Variables included in the model predicting preeclampsia were HbA1c at inclusion (OR 17 per % increase in HbA1c, 95 % CI 2.4-115, p = 0.004), preeclampsia in previous pregnancy (OR 27, 95 % CI 2.0-353, p = 0.01) and nulliparity (OR 7.7, 95 % CI 0.97-62, p = 0.054), see Table 2. The area under the ROC curve of this combination of variables in diagnosing preeclampsia was 0.79 (95 % CI 0.67-0.90), and 0.65 (95 % CI 0.48-0.82) for HbA1c alone. Six participants had HbA1c of 4.7 % (28 mmol/mol) or less and none of them had preeclampsia (this was the highest cut-off level giving 100 % sensitivity). Two participants had values higher than 5.7 % (39 mmol/mol), and both had preeclampsia (this was the lowest value giving 100 % specificity). Using these cut-off levels could have made us predict or exclude preeclampsia in eight of 228 women (3.5 %, 95 % CI 1.5-6.9 %). Neither GDM-WHO nor GDM-IADPSG diagnosed in first trimester was statistically significantly associated with preeclampsia.

Variables included in the model for birth weight were GDM-WHO in first trimester (β -311 g, 95 % CI -608 to -14, p = 0.04), using metformin at conception (β 255 g, 95 % CI 75-436, p = 0.006) and nulliparity (β -211 g, 95 % CI -381 to -41, p = 0.02), see Table 4. When substituting GDM-WHO in first trimester with GDM-IADPSG in first trimester the following variables were included in the model; HbA1c in first trimester (β -318 g per % increase in HbA1c, 95 % CI -660 to 25, p = 0.07), using metformin at conception (β 245 g, 95 % CI 66-423, p = 0.007) and nulliparity (β -193 g, 95 % CI -362 to -24, p = 0.03).

**Table 4:** Regression coefficients, 95 % confidence intervals and p-values for two models predicting birth weight (in grams)

Predictor	Model 1			Model 2		
	β	95 % CI	*p*-value	β	95 % CI	*p*-value
GDM-WHO in first trimester	-311	-608 to -14	0.04			
Using metformin at conception	255	75-436	0.006	245	66-423	0.007
Nulliparity	-211	-381 to -41	0.02	-193	-362 to -24	0.03
HbA1c				-318	-660 to 25	0.07

We did not find any non-linear association between the possible continuous predictor variables and the outcome variables.

## Discussion

HbA1c in first trimester could not discriminate well between GDM-WHO and NGT-WHO throughout pregnancy in PCOS women; only 2.2 % could have avoided an OGTT if we had required 100 % sensitivity and 100 % specificity to exclude and to predict GDM, respectively. When lowering the cut-offs to 96 % sensitivity and 94 % specificity, 15.8 % could have avoided an OGTT. However, more than one third of those avoiding an OGTT would be incorrectly classified. This was also true for GDM-WHO and GDM diagnosed by *modified* IADPSG criteria in first trimester only. However, first trimester HbA1c was statistically significantly associated with preeclampsia, a complication that the GDM diagnosis is supposed to predict, but could not predict in this study.

To our knowledge, this is the first study on HbA1c as a screening test for GDM in pregnant women with PCOS, who have a significantly increased risk of GDM, compared to an average pregnant population [17–20]. Several studies have indicated that HbA1c is not a suitable screening test for GDM, but most of them have measured HbA1c in late second or third trimester [4–8, 10]. Maegawa et al. evaluated HbA1c in first trimester as a screening test for GDM [9]. The population consisted of pregnant women in Japan. They used the GDM criteria of the Japan Society of Obstetrics and Gynecology and the Japan Diabetes Society, where two or more of the following results had to be present to diagnose GDM: Fasting glucose ≥ 5.6 mmol/L, 1-h glucose after 75-g OGTT ≥ 10.0 mmol/L and 2-h glucose after 75-g OGTT ≥ 8.3 mmol/L [9]. Their conclusion, that first trimester HbA1c was not suitable as a screening test for GDM diagnosed by glucose-based criteria, is in line with our present observation in PCOS women. Further, Hughes et al. found an area under the ROC curve for first trimester HbA1c in detecting GDM diagnosed by IADPSG OGTT criteria before gestational week 20 of only 0.711 [31]. An HbA1c < 4.8 % (29 mmol/mol) excluded GDM, however, the specificity was only 3.0 %. The study took place in a primary care setting in a population with a relatively low risk of GDM.

WHO states that women with hyperglycemia detected in pregnancy are at increased risk of adverse pregnancy outcomes, especially preeclampsia and macrosomia, and that treating GDM is effective in reducing adverse pregnancy outcomes [1]. In other words, a major goal in diagnosing GDM is to identify those at risk of pregnancy complications. According to our results, GDM-WHO in first trimester and HbA1c in first trimester were both negatively associated with birth weight, while the GDM-IADPSG diagnosis in first trimester was not associated with birth weight. It is noteworthy that the associations were in the opposite direction from what we expected, i.e. higher HbA1c and GDM-WHO in first trimester were associated with lower birth weight. Further, we found that HbA1c in first trimester was significantly associated with preeclampsia, while the GDM-WHO and the GDM-IADPSG diagnoses in first trimester were not. The OR for preeclampsia per % increase in HbA1c was as high as 17, but due to a relatively low number of study participants the uncertainty of the estimate was large (95 % CI 2.4-115). The HAPO (Hyperglycemia and Adverse Pregnancy Outcome) study concluded that for plasma glucose and HbA1c the ORs were similar for primary cesarean section, preeclampsia and preterm delivery [32]. These results are not in line with the present study, but in the HAPO study, glucose and HbA1c were measured during gestational week 24-32, which may explain some of the discrepancy. Another possible explanation is that we studied only pregnant women with PCOS. One study examining whether first trimester HbA1c could detect women at increased risk of preeclampsia, was the previously mentioned study by Hughes et al [31]. They found that the relative risk for preeclampsia was 2.42 (95 % CI 1.34-4.38) for women with a first trimester HbA1c of 5.9-6.4 % (41-46 mmol/mol) compared to those with HbA1c < 5.9 %. However, no results on first trimester plasma glucose or GDM in predicting preeclampsia were reported, so we cannot compare HbA1c to any of those.

In several of the diagnostic criteria for GDM used today, including the WHO and IADPSG criteria, only one plasma glucose value above a diagnostic cut-off limit is required to diagnose GDM [2]. The reproducibility of OGTT in pregnancy, measured with 1-2 weeks gap, has been reported to be poor; 22-24 % of the participants were reclassified [33, 34]. Considering the results of Hughes et al. [31] (i.e. that HbA1c identifies women at increased risk of preeclampsia) and ours (i.e. that HbA1c might be a better predictor for preeclampsia than GDM diagnosis), the poor reproducibility of OGTT in pregnancy and advantages of HbA1c compared to OGTT (as fasting, glucose ingestion and timed samples is not required, and that the preanalytical instability is less), we suggest that HbA1c in first trimester should be further evaluated as a possible risk marker for preeclampsia and compared to GDM diagnosed by established criteria in larger, prospective studies in various populations.

Women with fasting plasma or serum glucose > 7.0 mmol/L at time of inclusion were not included in the study, so our results are not representative for that group of women. However, only one of the 348 women considered eligible for inclusion was not included due to a fasting plasma glucose >7.0 mmol/L. Further, only one woman had a plasma glucose result at the time she was diagnosed with GDM-WHO above the diagnostic threshold for diabetes mellitus in non-pregnant women [24]. She had elevated fasting plasma glucose in gestational week 36. Our results are not representative for pregnant women with glucose values consistent with diabetes mellitus in non-pregnant adults. In the study by Hughes et al. the area under the ROC curve for first trimester HbA1c in detecting diabetes diagnosed by WHO OGTT criteria before 20 weeks gestation was 0.991, with a sensitivity of 100 % and specificity of 97.4 % at the optimal cut-off point of 5.9 % (41 mmol/mol) [31]. Accordingly, one could expect that first trimester HbA1c performs better as a screening test for higher degrees of glycemia than in our study, also in pregnant women with PCOS.

About one third of the women in the present study used metformin at conception and in early pregnancy, followed by a washout period of at least 7 days before inclusion in the study (Table 1). Further, half of the participants were randomised to metformin from first trimester to delivery (Table 1). Seventy-nine women (35 %) did not use metformin at any time point during pregnancy. GDM prevalence and main characteristics by metformin use and GDM status can be found in Additional file 1. As metformin is an antidiabetic drug, one could suspect that the use of metformin might have influenced our results. Currently, the only randomised controlled trial on women with PCOS on metformin versus placebo from first trimester to delivery with GDM as one of the outcome measures is the PregMet study, which the present results are from. In the PregMet study, metformin did not demonstrate an effect on the prevalence of GDM [35]. Further, in the pilot study to the PregMet study, no effect of metformin on indices of glucose homeostasis in women with PCOS was found [36]. However, this does not exclude that metformin has an effect during pregnancy, and especially, it says nothing about the effect of metformin at conception and early in pregnancy. Because of this, randomisation was considered as an independent variable in the models predicting GDM throughout pregnancy, preeclampsia and birth weight. Using metformin after randomisation did not reach the significance level required to be included in a model (p ≤ 0.10) for any of the models. Further, using metformin at conception and in early pregnancy was also considered as an independent variable for all the models in the present study, in order to adjust for possible confounding. It only reached the significance level required to be included in a model (p ≤ 0.10) for the models predicting birth weight.

Those diagnosed with GDM-WHO in the present study received more thorough diet and lifestyle advice compared to those with NGT-WHO, and two were treated with insulin, and could thereby have prevented adverse outcomes. By this, it is possible that the associations between the predictor variables and the adverse pregnancy outcomes are underestimated. Further, we were only able to evaluate HbA1c as a screening test related to *modified* IADPSG criteria as 1-h glucose values from OGTT were not available. Studies using *modified* IADPSG criteria have already been published [37, 38]. The proportion of GDM cases diagnosed by 1-h value only when using the IADPSG criteria, has been estimated to 14-21 % [39–41]. According to this, we might have lost up to one fifth of GDM-IADPSG cases, and this must be kept in mind when interpreting the results. In addition, we could evaluate HbA1c as a screening test for GDM diagnosed by the *modified* IADPSG criteria in first trimester only, and not later in pregnancy. The reason for this was that those with a GDM diagnosis by the WHO criteria did not perform an OGTT at later time points, i.e. only those not diagnosed with GDM-WHO performed OGTT at later time points. As an example if a woman got GDM by WHO criteria because of a 2-h serum glucose value of 8.0 mmol/L and fasting plasma glucose of 5.0 mmol/L, she would not get the diagnosis by IADPSG criteria, and we would not know if she would have developed GDM by IADPSG criteria at a later time point. Accordingly, we cannot tell whether first trimester HbA1c could be a potential screening test for GDM diagnosed by IADPSG criteria later in pregnancy.

## Conclusion

In women with PCOS, first trimester HbA1c is not a good screening test for GDM in the first trimester or later in pregnancy; however, it might be better than the GDM diagnosis in predicting preeclampsia.

## Additional file

Additional file 1:
**Main characteristics of study participants by metformin use and GDM status. Numbers are median (min-max) or N (%).**

## Abbreviations

CI: Confidence interval
GDM: Gestational diabetes mellitus
HAPO: Hyperglycemia and adverse pregnancy outcome
HbA1c: Glycated hemoglobin A1c
IADPSG: The International Association of the Diabetes and Pregnancy Study Groups
NGT: Normal glucose tolerance
OGTT: Oral glucose tolerance test
OR: Odds ratio
PCOS: Polycystic ovary syndrome
ROC: Receiver operating characteristic
WHO: World Health Organization
