# Supplementary material for: HbA1c as screening for gestational diabetes mellitus in women with polycystic ovary syndrome
Source: BMC Endocr Disord. 2015 Aug 6;15:38. doi: 10.1186/s12902-015-0039-9 (PMC4527320; doi:10.1186/s12902-015-0039-9)
Supplement: Additional file 1: — Main characteristics of study participants by metformin use and GDM status. Numbers are median (min-max) or N (%). [file 12902_2015_39_MOESM1_ESM.pdf]

	Using metformin at conception				Randomised to metformin				Using metformin at conception and/or randomised to metformin			
	Yes		No		Yes		No		Yes		No	
Characteristic	GDM-WHO N=18 (25%)	NGT-WHO N=55	GDM-WHO N=37 (24%)	NGT-WHO N=118	GDM-WHO N=28 (25%)	NGT-WHO N=86	GDM-WHO N=27 (24%)	NGT-WHO N=87	GDM-WHO N=37 (25%)	NGT-WHO N=112	GDM-WHO N=18 (23%)	NGT-WHO N=61
Age (years)	31 (25-40)	29 (20-38)	30 (22-44)	29 (19-39)	31 (23-44)	29 (19-39)	31 (22-36)	29 (20-39)	31 (23-44)	29 (19-39)	31 (22-36)	29 (21-39)
BMI (kg/m^2)	29.1 (22.6-45.7)	27.3 (18.8-46.2)	28.9 (19.6-44.2)	26.2 (19.0-50.2)	28.2 (20.1-44.2)	27.5 (19.0-50.2)	29.8 (19.6-45.7)	25.9 (18.8-44.8)	29.8 (20.1-45.7)	27.2 (18.8-50.2)	28.6 (19.6-43.9)	25.8 (19.0-44.8)
HbA1c (%)	5.2 (4.7-6.3)	5.1 (4.7-5.6)	5.1 (4.9-6.6)	5.1 (4.6-5.6)	5.3 (4.8-6.3)	5.2 (4.7-5.6)	5.1 (4.7-6.6)	5.1 (4.6-5.6)	5.2 (4.7-6.3)	5.2 (4.7-5.6)	5.1 (4.9-6.6)	5.1 (4.6-5.6)
Birth weight (g)	3590 (2995-4730)	3702 (2275-4750)	3405 (165-4150)	3520 (1530-4840)	3593 (2330-4730)	3520 (1530-4520)	3410 (165-4480)	3734 (1840-4840)	3580 (2330-4730)	3555 (1530-4750)	3175 (165-3960)	3728 (1840-4840)
Nulliparity (no.)	7 (39)	38 (69)	21 (57)	66 (56)	16 (57)	51 (59)	12 (44)	53 (61)	18 (49)	70 (63)	10 (56)	34 (56)
	GDM-WHO first trimester N=10 (14%)	NGT-WHO first trimester N=62	GDM-WHO first trimester N=10 (6.5%)	NGT-WHO first trimester N=144								
Age (years)	32 (25-40)	31 (20-40)	33 (25-44)	29 (19-39)								
BMI (kg/m^2)	31.7 (24.7-45.7)	27.0 (18.8-46.2)	30.7 (24.2-40.4)	26.7 (19.0-50.2)								
HbA1c (%)	5.3 (4.7-6.3)	5.1 (4.7-5.6)	5.2 (4.9-6.6)	5.1 (4.6-5.6)								
Birth weight (g)	3716 (3540-4730)	3668 (2275-4750)	3088 (165-4150)	3500 (1530-4840)								
Nulliparity (no.)	4 (40)	40 (65)	5 (50)	82 (57)								
	GDM-IADPSG first trimester N=16 (22%)	NGT-IADPSG first trimester N=56	GDM-IADPSG first trimester N=19 (12%)	NGT-IADPSG first trimester N=135								
Age (years)	31 (22-36)	31 (20-40)	32 (23-44)	29 (19-39)								
BMI (kg/m^2)	29.0 (18.8-45.7)	28.1 (19.9-46.2)	29.8 (20.0-40.5)	26.6 (19.0-50.2)								
HbA1c (%)	5.1 (4.7-6.3)	5.2 (4.7-5.7)	5.0 (4.9-6.6)	5.1 (4.6-5.6)								
Birth weight (g)	3570 (3236-4730)	3720 (2275-4750)	3495 (414-4280)	3470 (165-4840)								
Nulliparity (no.)	11 (69)	33 (59)	11 (58)	76 (56)								

Appendix Table 5. Main characteristics of study participants by metformin use and GDM status. Numbers are median (min-max) or N (%).
